# Microbiome Profile of Dogs with Stage IV Multicentric Lymphoma: A Pilot Study

**DOI:** 10.3390/vetsci9080409

**Published:** 2022-08-04

**Authors:** Feriel Yasmine Mahiddine, Inhwan You, Heekee Park, Min Jung Kim

**Affiliations:** Department of Research and Development, Mjbiogen Corp., Seoul 04788, Korea or or or

**Keywords:** lymphoma, gut microbiome, canine

## Abstract

**Simple Summary:**

Lymphoma is a common type of hematopoietic cancer encountered in small animal practices. Canine multicentric lymphoma represents 80% of lymphoma cases and is characterized by a spread of the disease in multiple lymph nodes and organs as well. A causal role of the gut microbiota in disease spread has been shown in different diseases. In this study, the gut microbiome of dogs diagnosed with stage IV multicentric lymphoma has been analyzed and compared with that of healthy dogs to evaluate potential changes linked to lymphoma and disease spread.

**Abstract:**

Changes in the gut microbiome can be associated with diseases and affect the overall health of an individual. In the current study, the gut microbiome profile of dogs diagnosed with advanced stages of multicentric lymphoma was compared with that of healthy dogs and analyzed. For this purpose, dogs from veterinary hospitals diagnosed with lymphoma were selected and were further narrowed down to cases of stage IV multicentric lymphoma. Fecal samples from the selected sick and healthy dogs were collected and analyzed using MiSeq sequencing. The gut microbiota in the two groups of dogs was statistically analyzed and compared. The results revealed significant differences in the microbial populations present in sick and healthy dogs. Phylum Actinobacteria and two species (*Corynebacterium amycolatum* and *Streptococcus lutetiensis*) were found in high proportions in sick dogs and may be considered as potential biomarkers for canine stage IV multicentric lymphoma. Further investigations need to be conducted to understand the mechanisms they might be involved in.

## 1. Introduction

Fecal microbiome analysis is commonly used to predict gastrointestinal bacterial composition because feces are easily accessible for sampling [1]. Although the fecal microbiome is not a representative sample of the whole gut microbiome, it could be useful in understanding certain health conditions. For instance, obesity is often associated with a perturbation in the Firmicutes/Bacteroidetes ratio [2,3]. A dysbiosis in the gut microbiome can lead to the onset or progression of many diseases because gut microorganisms play a major role in protection against pathogens [4]; they are also associated with immunomodulatory and anticancer activities [5,6]. *Faecalibacterium* [7], *Fusobacterium*, *Clostridium hiranonis*, *Blautia,* and *Turicibacter* usually decrease in dysbiosis; whereas, *Streptococcus* and *Escherichia coli* populations increase [8]. Diseases can initiate gastrointestinal dysbiosis, which is evident in fecal microbiome samples of sick individuals, and have a stronger influence on gut microbial populations than other factors such as breed, environment, and nutrition [9]. Recent studies have focused on biomarker development by comparing the composition of the gut microbiome of healthy and sick individuals to predict or diagnose certain diseases [10,11,12].

Lymphoma is the most common hematopoietic cancer in dogs, with an annual incidence of 20–100 cases per 100,000 dogs [13]. Immunophenotyping and evaluation of clinical stages are essential prognostic factors for diagnosing lymphoma. The most commonly diagnosed lymphoma is diffuse large B-cell lymphoma (DLBCL) with an incidence of 40% among canine lymphoma cases [14,15]. DLBCL is the most common multicentric lymphoma and is characterized by a survival span of approximately a year, depending on the stage and the treatment protocol. Recent evidence indicates that gut microbiota populations are modulated by various diseases, including cancer [16]. Considering that the gastrointestinal tract is a common site for primary extranodal lymphoma, studying the changes in gut microbiota could reveal its potential role in cancer progression and associated pathognomonic changes as well as the differences between the different types of lymphoma and their influence on the gut microbiome. Moreover, since microbiota composition can impact the efficiency of cancer treatments [17], screening the microbial populations present in individuals with multicentric lymphoma can help predict the outcome of therapy. In the present study, the gut microbiota of dogs with stage IV multicentric lymphoma was compared with that of healthy dogs and analyzed.

## 2. Materials and Methods

### 2.1. Animal Use and Clinical Data Collection

Healthy dogs and dogs diagnosed with lymphoma from several veterinary hospitals in South Korea were used in the present study, with consent from the dog owners. Healthy dogs that underwent regular health check-ups were selected based on their clinical history and the results of their check-up: absence of disease, inflammation, gastrointestinal problems, and chronic conditions. Healthy dogs that were on medications or dietary supplements were excluded from the study. Lymphoma diagnosis was confirmed by a veterinary pathologist. Dogs diagnosed with multicentric lymphoma were selected based on the type of lymphoma, stage, substage, clinical history (including the presence or absence of gastrointestinal problems), and medication history. The World Health Organization (WHO) classification was used for the grading of lymphoma [18], as shown in Table 1. Fecal samples of dogs with stage IV multicentric lymphoma that have not started chemotherapy or any other treatment were chosen for the lymphoma (LM) group, and fecal samples from healthy dogs (H) group that underwent regular health check-ups were used as the control group.

### 2.2. Sampling and Sequencing Analysis

Rectal swab samples were collected from dogs diagnosed with lymphoma and healthy dogs using N-SWAB TRANSPORT (Noble Bio, Hwaseong, Korea) at veterinary clinics. Samples were stored at −80 °C, and microbial genomic DNA was extracted using the DNeasy PowerSoil Kit (Qiagen, Hilden, Germany). 16S rRNA sequences were analyzed as previously described [19]. In brief, the Illumina 16S Metagenomic Sequencing Library Prep Guide was used for the V3-V4 region. Paired-end sequencing was done on the MiSeq™ platform (Illumina, San Diego, CA, USA) by Macrogen (Seoul, Korea). The resulting trimmed sequences were clustered into operational taxonomic units (OTUs) based on 97% identity/similarity, and microbial community analysis was performed using Quantitative Insights Into Microbial Ecology (QIIME) 1.9 [20].

### 2.3. Statistical Analysis

Beta diversity was measured using unweighted and weighted UniFrac distances and visualized using principal coordinate analysis (PCoA) for comparison between the microbial compositions of the two groups. The boxplot package in R v3.0.1 was used for the rest of the analyses. The Mann–Whitney test was used to compare the microbial diversity indices and relative abundances in the two groups to determine significant variations between them. All values were expressed as mean ± standard error of the mean (SEM), and all *p* values < 0.05 were considered statistically significant.

## 3. Results

### 3.1. Study Population

Among the 13 dogs diagnosed with lymphoma, 10 were found to have multicentric lymphoma, and only 9 were diagnosed with stage IV multicentric substage-a lymphoma. Among those 9 dogs, 2 were undergoing chemotherapy; therefore, they were excluded from the study. In total, 7 dogs were selected for the LM group. In the H group, among 30 dogs that came for a regular check-up, only 11 dogs were evaluated to be healthy dogs and selected for the study. The average age of the H group was 5.5 ± 1.1 years (*n* = 11) and the average age of the LM group was 8.6 ± 2.0 years (*n* = 7). The breeds and sex of the selected dogs for the H group were as follows: Maltese (*n* = 5), Chihuahua (*n* = 2), English cocker spaniel (*n* = 1), Yorkshire terrier (*n* = 1), toy poodle (*n* = 1), and Welsh corgi (*n* = 1); castrated males (*n* = 6), spayed females (*n* = 4), and intact females (*n* = 1). For the LM group, the breeds and sex of the selected dogs were as follows: Labrador retriever (*n* = 1), cocker spaniel (*n* = 2), Yorkshire terrier (*n* = 2), mixed breed (*n* = 1), and dachshund (*n* = 1); castrated males (*n* = 5), spayed females (*n* = 1), and intact males (*n* = 1).

### 3.2. Lymphoma Type Classification

The diagnosis and staging of lymphoma cases were confirmed using ultrasonography (*n* = 6), polymerase chain reaction-based clonality testing (*n* = 2), computerized tomography scan (*n* = 1), radiography (*n* = 6), cytology (*n* = 7), and blood tests (*n* = 6). The LM group dogs exhibited generalized lymphadenopathy.

### 3.3. Gut Microbiome Analysis

**Figure 1:**
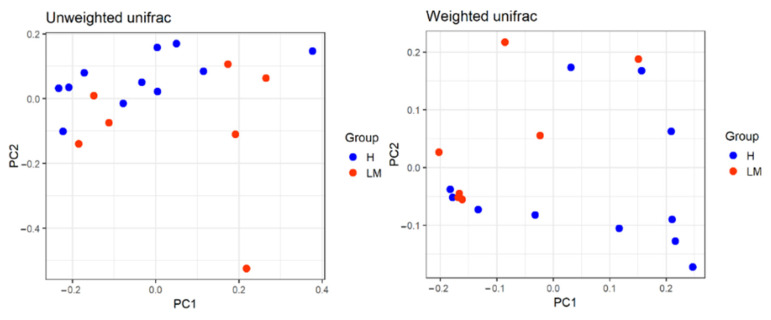
Beta diversity using weighted and unweighted UniFrac distances between healthy (H) and lymphoma (LM) groups.

**Figure 2:**
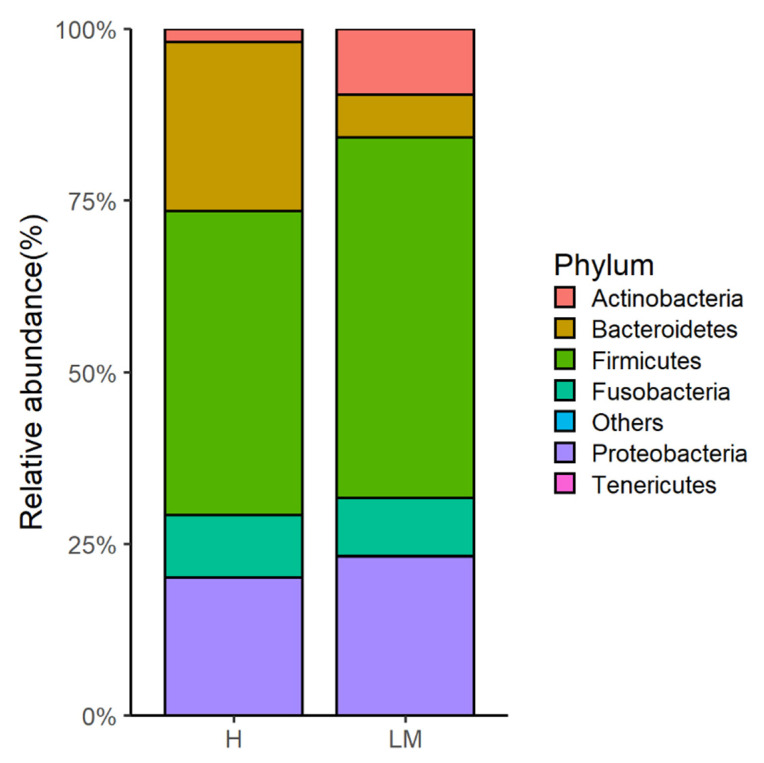
Relative abundance at the phylum level between healthy (H) and lymphoma (LM) dogs.

Alpha diversity results revealed no significant difference between the two groups as shown in Table 2. Beta diversity was represented with PCoA plots based on weighted and unweighted UniFrac distances; both showed distinctive clustering between the two groups (Figure 1). Gut microbiome analysis revealed a significantly higher abundance of Actinobacteria in the LM group than in the H group results (9.6 ± 3.7% vs. 1.9 ± 0.7%), as shown in Figure 2. However, Bacteroidetes were nearly four times significantly less abundant in the LM group samples than in the H group samples (6.2 ± 4.1% vs. 24.6 ± 6.4%). Firmicutes increased in the LM group but demonstrated no significant difference in the H group. Among the genera with an abundance higher than 1%, two species showed a significant difference in population in the LM group and H group: *Corynebacterium* was significantly higher in the LM group than in the H group (8.3 ± 3.1% vs. 1.2 ± 0.5%) and *Kineothrix* was significantly lower in the LM group than in the H group (0.7 ± 0.6% vs. 1.4 ± 0.3%). The species with an abundance higher than 1% were *Corynebacterium* *amycolatum, Blautia schinkii, Clostridium spiroforme,* and *Kineothrix alysoides* as shown in Table 3.

## 4. Discussion

Diseases can influence fecal microbiota composition and induce gastrointestinal dysbiosis. Studies on the gut microbiota composition in diseased organisms are needed to identify changing patterns that could be pathognomonic of certain conditions. Lymphoma is the most common hematopoietic tumor in dogs, with an annual incidence of 20–100 per 100,000 dogs; however, this incidence is gradually increasing [21]. Lymphomas can be categorized into multicentric, alimentary, and extranodal lymphoma [22]. Among them, multicentric lymphoma accounts for 75–85% of canine lymphoma cases and is the most common type [23,24]. However, the number of studies on gut microbiome changes associated with canine multicentric lymphoma, with and without CHOP chemotherapy, is limited [25,26]. In addition, although canine multicentric lymphoma is divided into stages (I to V) and substages (a or b), previous studies at different stages were limited by factors such as the stage difference and disease progression, treatments, and the presence of intestinal symptoms [25,26,27]. Considering the diversity in disease stages and interindividual variations, standard values, and changing patterns associated with lymphoma, in the present study, we focused on cases pertaining to stage IV multicentric lymphoma; the fecal microbiome results of the LM group of dogs were compared with those of the H group of dogs.

The predominant bacterial phyla in both groups were Actinobacteria, Bacteroidetes, Firmicutes, Fusobacteria, and Proteobacteria. The relative abundance of Actinobacteria was significantly higher in the LM group than in the H group; whereas Bacteroidetes was almost four times lower in the LM group than in the H group. Actinobacteria are essential for gut barrier homeostasis and phylum diversity [28], and their relative abundance fluctuates depending on the cause of dysbiosis. Few studies have recorded reduced levels of Actinobacteria in dogs with chronic kidney disease and Sjögren’s syndrome, a disorder of the immune system; whereas there is a significant increase in Actinobacteria levels in dogs with gastric cancer and canine inflammatory bowel disease [9,29,30]. In the present study, Actinobacteria significantly increased in dogs with stage IV multicentric lymphoma. These findings suggest that Actinobacteria proportions increase in cases of cancer and gastrointestinal pathologies in dogs.

Furthermore, some species of Actinobacteria possess antioxidant activity or genes that encode antioxidant enzymes [31,32]. Previous studies have demonstrated that antioxidants promote cancer cell survival during extra-cellular matrix detachment which facilitates cancer progression and metastasis [33]. Therefore, we hypothesize that the high proportions of Actinobacteria in the gut may play a vital role in lymphoma progression through antioxidant activity; specifically, species from this phylum were present at significantly higher levels than others, such as *Corynebacterium amycolatum*. Additionally, serum antioxidant levels in stages III-IV of canine multicentric lymphoma (substages a and b) increase, which was measured by the ferric reducing ability of plasma (FRAP) assay [34]. Lymphoma stages III and IV are characterized by the spread of cancer to other organs; therefore, the role of antioxidants in lymphoma progression must be investigated. Similarly, reduced Bacteroidetes levels are associated with gut dysbiosis, leading to the inhibition and proliferation of certain species [35]. Additionally, Bacteroidetes are responsible for producing short-chain fatty acids (SCFAs) which are considered anticarcinogenic [36]. Reduced levels of SCFAs can facilitate disease progression because they possess immunomodulatory effects. These effects are particularly observed in B-type lymphocytes because SCFAs can regulate B-cell activity in B-cell malignancies and prevent the B-cell class from switching into IgA, IgD, IgG, or IgM [37]. Moreover, SCFAs can repress *BCL-6* [38], a proto-oncogene that plays a vital role in the development of lymphomagenesis [39]. A decrease in the proportion of Bacteroidetes may be a factor in the progression of lymphoma in stage IV cases. However, these results are from fecal sample analysis only, and the intestinal mucosal surface has an important role in maintaining the host-bacterial symbiosis through the interaction between the mucosal microbiota populations and the intestinal immune system. The effects of lymphoma on the populations residing at the epithelial-lumen interface need to be studied as well. In summary, the fecal microbiota analysis revealed that multicentric lymphoma induces gut dysbiosis in dogs, which may lead to lymphoma progression.

The genus and species populations results obtained in the present study differed from those obtained in previous studies conducted on fecal microbiota populations of canine multicentric lymphoma cases. While previous studies found decreased proportions of *Fusobacterium*, *Turicibacter*, *Blautia*, and *Faecalibacterium* in dogs with lymphoma [25,26], our results showed decreased populations of *Kineothrix*, *Caproiciproducens*, and *Roseburia*. Moreover, the relative abundance of *Turicibacter* and most of the *Blautia* species was higher in the fecal samples of the LM group than in those of the H group, as shown in Tables S1 and S2. Additionally, in human patients, *Roseburia* was previously found in higher abundance in the H group than in the LM group [40]. *Roseburia* is also an SCFA producer [41] and, similar to our results, a previous study found decreased levels of *Roseburia* to be associated with microbial dysbiosis in colorectal carcinogenesis [42]. Because *Roseburia*-associated intestinal butyrate production is linked with a reduced incidence of colon cancer [43], its decrease can also be a decisive factor in lymphoma progression. The genera that showed an increase in our results were also different from those found in other studies; the relative abundance of *Corynebacterium*, *Peptostreptococcus*, and *Proteus* was higher in the LM group than in the H group. These differences in the fecal microbiota populations in sick dogs in previous studies and our study indicate that the disease stage may influence the gut microorganism populations by favoring populations that promote disease progression. Other factors such as diet, housing, breed, and physical activity could also influence the microbiota composition and should be studied separately. However, in this study and in accordance with previous results, disease seems to be a stronger factor in modulating gut microbiota populations [9].

Although not statistically significant, our results also found *Streptococcus* in a higher proportion in the LM group than in the H group [25,26]. Specifically, at the species level, *Streptococcus lutetiensis* abundance was high in the LM group without any statistical significance, whereas *Streptococcus fryi* level was found to be significantly different in the two groups, but its relative abundance was not significantly higher in the LM dogs than in H group. Little is known about *S. fryi* and its role in the gut microbiome of dogs and other species. Furthermore, *S. lutetiensis* was recently isolated for the first time from a cat with intestinal lymphoma [44]. The authors of that study suggested that the impact of intestinal lymphoma on host immunity could influence *S. lutetiensis* replication in cats. This same species was also found in patients with diarrhea of unknown etiology [45] and is suspected to be involved in colorectal cancer carcinogenesis [46]. In the present study, *S. lutetiensis* was higher in the LM group with no intestinal symptoms than in the H group. Therefore, it is indicated that *S. lutetiensis* may be involved in carcinogenesis or disease progression rather than in the onset of intestinal symptoms.

At the species level, *C. amycolatum* was the only species that was four times more abundant in the LM group than in the H group. It is a Gram-positive bacillus and commensal bacterium found on dog and human skin [47]. It can be responsible for different types of infections ranging from bone and joint infections to endocarditis [48,49]. Moreover, this species has a higher antibiotic resistance than other similar *Corynebacterium* species [50] and is involved in ear infections in immunocompromised patients [51]. Our results indicate that higher levels of *C. amycolatum* could have a significant association with multicentric lymphoma in dogs, especially through its potential antioxidant capacity, as discussed previously. This is the first time this species was isolated from dogs with stage IV multicentric lymphoma. More studies are necessary to evaluate the relevance and role of this species in the onset and progression of the disease and the possible pathways involved.

## 5. Conclusions

The results of this study revealed that the composition of the gut microbiota fluctuates depending on the health condition of an individual. Stage IV multicentric lymphoma tends to influence the gut microbiome differently than the other stages, which were previously studied. Actinobacteria and Bacteroidetes populations are significantly different in sick and healthy dogs. Particularly, *S. lutetiensis* and *C. amycolatum* are four times higher in sick dogs than in healthy dogs. The changes and the differences found between the present study and previous studies emphasize the need for more targeted studies on the gut microbiota changes associated with different stages of canine multicentric lymphoma.

## Figures and Tables

**Table 1:** Lymphoma grading system in dogs according to WHO classification of tumors in domestic animals.

Disease Extent	Signs	Stage
Involve a limited area of the body	One group of lymph nodes A single body organ (extranodal lymphoma)	Stage I
	Two or more groups of lymph nodes in the front half or back half of the body Extranodal lymphoma and the presence of one or more groups of lymph nodes on the same side of the diaphragm	Stage II
Advanced disease	Generalized lymphadenopathy	Stage III
	Generalized lymphadenopathy with involvement of the liver, and/or spleen	Stage IV
	Stage I to IV with involvement of blood or bone marrow	Stave V
Substages	No systemic signs	A substage
	Unexplained weight loss Fever Night sweats Hypercalcemia	B Substage

**Table 2:** Alpha diversity indices in healthy (H) and lymphoma (LM) groups.

Index	H	LM	*p* Value
Observed species	78.5 ± 7.4	94.9 ± 12.7	0.389
Chao1	79.1 ± 7.4	96.6 ± 13.2	0.415
Shannon	3.7 ± 0.1	4.2 ± 0.3	0.239
Simpson	0.8 ± 0	0.9 ± 0	0.122

**Table 3:** Relative abundances of main bacterial groups (more than 0.1% proportion) in healthy (H) and lymphoma (LM) dogs at the phylum, genus, and species levels.

Bacteria	H	LM	*p* Value
Phylum			
Actinobacteria	1.9 ± 0.7	9.6 ± 3.7	0.024
Bacteroidetes	24.6 ± 6.4	6.2 ± 4.1	0.037
Genus			
*Corynebacterium*	1.2 ± 0.5	8.3 ± 3.1	0.018
*Kineothrix*	1.4 ± 0.3	0.7 ± 0.6	0.036
*Caproiciproducens*	0.3 ± 0.3	0.0 ± 0.0	0.032
*Peptostreptococcus*	0.0 ± 0.0	0.3 ± 0.2	0.010
*Proteus*	0.2 ± 0.1	0.6 ± 0.2	0.042
*Roseburia*	0.4 ± 0.3	0.0 ± 0.0	0.033
Species			
*Corynebacterium amycolatum*	0.5 ± 0.4	4.3 ± 1.5	0.016
*Blautia schinkii*	3.5 ± 1.4	0.0 ± 0.0	0.016
*[Clostridium] spiroforme*	1.5 ± 0.9	0.1 ± 0.1	0.042
*Kineothrix alysoides*	1.4 ± 0.3	0.7 ± 0.6	0.036
*Caproiciproducens galactitolivorans*	0.3 ± 0.3	0.0 ± 0.0	0.023
*Peptostreptococcus canis*	0.0 ± 0.0	0.3 ± 0.2	0.046
*Proteus mirabilis*	0.2 ± 0.1	0.6 ± 0.2	0.042
*Roseburia intestinalis*	0.4 ± 0.3	0.0 ± 0.0	0.033

## Data Availability

Data will be available on request from the first author.

## Supplementary Materials

The following supporting information can be downloaded at: https://www.mdpi.com/article/10.3390/vetsci9080409/s1, Table S1: Relative abundance of fecal microbiome genera present in healthy and sick dogs; Table S2: Relative abundance of fecal microbiome species present in healthy and sick dogs.
