# Supplementary material for: Microbiome Profile of Dogs with Stage IV Multicentric Lymphoma: A Pilot Study
Source: Vet Sci. 2022 Aug 4;9(8):409. doi: 10.3390/vetsci9080409 (PMC9414525; doi:10.3390/vetsci9080409)
Supplement: Supplementary file 1 [file vetsci-09-00409-s001.zip › vetsci-1808865-supplementary.pdf]

Table S1. Relative abundance of fecal microbiome genra present in healthy and sick dogs

Genus	Healthy group	LM group	*p* Value
Abyssivirga	0.2 ± 0.1	0.6 ± 0.4	0.216
Actinomyces	0 ± 0	0.1 ± 0.1	0.295
Allisonella	0 ± 0	0 ± 0	0.254
Amedibacillus	0.5 ± 0.2	0.9 ± 0.4	0.272
Anaerobacterium	0 ± 0	0 ± 0	0.78
Anaerobiospirillum	0 ± 0	0 ± 0	0.494
Anaerostipes	0.1 ± 0.1	0 ± 0	0.119
Anaerotignum	0.1 ± 0.1	0 ± 0	0.813
Arcanobacterium	0 ± 0	0.1 ± 0.1	0.254
Bacteroides	6.6 ± 3.3	2 ± 0.9	0.618
Beduini	0.1 ± 0.1	0 ± 0	0.78
Bifidobacterium	0.3 ± 0.2	0 ± 0	0.093
Blautia	10.6 ± 3.6	10.8 ± 4.2	1
Buchananella	0 ± 0	0 ± 0	0.007
Bulleidia	0 ± 0	0 ± 0	0.494
Butyribacter	0 ± 0	0 ± 0	0.494
Butyricicoccus	0.5 ± 0.3	1 ± 0.9	0.927
Campylobacter	0.1 ± 0.1	0.1 ± 0.1	0.619
Canicola	0 ± 0	0.2 ± 0.2	0.295
Caproiciproducens	0.3 ± 0.3	0 ± 0	0.023
Catenibacterium	0 ± 0	0 ± 0	0.494
Cellulosilyticum	0 ± 0	0 ± 0	0.214
Citrobacter	0 ± 0	0.6 ± 0.6	0.295
Clostridioides	1.2 ± 0.5	2.2 ± 1.3	0.891
Clostridium	3.3 ± 1.8	5.2 ± 2.5	0.618
Collinsella	0.4 ± 0.2	0 ± 0	0.456
Coprobacter	0 ± 0	0.1 ± 0.1	0.254
Corynebacterium	1.2 ± 0.5	8.3 ± 3.1	0.018
Desulfovibrio	0 ± 0	0 ± 0	0.74
Dialister	0 ± 0	0 ± 0	0.494
Eggerthella	0 ± 0	0.2 ± 0.2	0.842
Enterobacter	0 ± 0	0 ± 0	0.868
Enterocloster	0 ± 0	0.1 ± 0	0.551
Enterococcus	0.3 ± 0.1	2.8 ± 1.6	0.134
Erysipelatoclostridium	2.8 ± 1	0.9 ± 0.4	0.205
Escherichia	15.2 ± 4.7	19.3 ± 6	0.365
Faecalibacterium	0.1 ± 0.1	0 ± 0	0.78

Faecalimonas	4.3 ± 1.9	2.9 ± 1	1
Finegoldia	0 ± 0	0.5 ± 0.4	0.265
Flavonifractor	0.2 ± 0.1	0.2 ± 0.1	0.236
Flintibacter	0 ± 0	0 ± 0	0.517
Fournierella	0 ± 0	0 ± 0	0.28
Frederiksenia	0 ± 0	0 ± 0	0.494
Fusobacterium	9.1 ± 4.4	8.4 ± 4.3	0.923
Gemella	0 ± 0	0 ± 0	0.254
Gleimia	0 ± 0	0 ± 0	0.082
Gluceribacter	2.3 ± 0.6	0.9 ± 0.8	0.118
Haemophilus	0 ± 0	0 ± 0	0.254
Helicobacter	3.7 ± 3.7	0.5 ± 0.5	0.663
Kineothrix	1.4 ± 0.3	0.7 ± 0.6	0.036
Klebsiella	0.1 ± 0.1	0.4 ± 0.3	0.164
Lacrimispora	0 ± 0	0 ± 0	0.494
Lactobacillus	0 ± 0	0 ± 0	0.494
Lactonifactor	0 ± 0	0 ± 0	0.254
Lawsonella	0 ± 0	0.2 ± 0.2	0.254
Leuconostoc	0 ± 0	0 ± 0	0.254
Ligilactobacillus	0 ± 0	0.3 ± 0.3	0.254
Massilimicrobiota	0 ± 0	0.1 ± 0.1	0.74
Mediterranea	0.9 ± 0.9	0 ± 0	0.494
Mediterraneibacter	5.1 ± 1.2	9 ± 4.1	0.786
Megamonas	1.8 ± 1.6	0 ± 0	0.534
Megasphaera	0 ± 0	0 ± 0	0.494
Mitsuokella	0 ± 0	0 ± 0	0.254
Morganella	0 ± 0	0.2 ± 0.2	0.265
Natranaerovirga	0 ± 0	0 ± 0	0.254
Novisyntrophococcus	0 ± 0	0 ± 0	0.889
Odoribacter	0 ± 0	0 ± 0	0.494
Paeniclostridium	0.2 ± 0.1	0.4 ± 0.3	0.371
Parabacteroides	5.4 ± 4.3	0.2 ± 0.2	0.909
Paraprevotella	0 ± 0	0 ± 0	0.494
Parasutterella	0.1 ± 0.1	0 ± 0	0.494
Pasteurella	0 ± 0	0.2 ± 0.2	0.254
Peptacetobacter	2.7 ± 2.6	0 ± 0	0.78
Peptoniphilus	0 ± 0	0.2 ± 0.2	0.265
Peptostreptococcus	0 ± 0	0.3 ± 0.2	0.01
Phascolarctobacterium	0.1 ± 0.1	0 ± 0	0.78
Phocaeicola	11.2 ± 5.9	1.1 ± 0.7	0.333
Phocea	0 ± 0	0.1 ± 0.1	0.295

Porphyromonas	0 ± 0	2.7 ± 2.7	0.494
Prevotella	0 ± 0	0 ± 0	0.254
Prevotellamassilia	0.4 ± 0.4	0 ± 0	0.494
Proteus	0.2 ± 0.1	0.6 ± 0.2	0.042
Pseudoflavonifractor	0 ± 0	0 ± 0	0.163
Pseudomonas	0 ± 0	0 ± 0	0.026
Romboutsia	0.1 ± 0	0.9 ± 0.4	0.162
Roseburia	0.4 ± 0.3	0 ± 0	0.033
Ruthenibacterium	0 ± 0	0 ± 0	0.254
Schaalia	0 ± 0	0.1 ± 0.1	0.059
Sellimonas	0 ± 0	0 ± 0	0.281
Shigella	0.4 ± 0.2	0 ± 0	0.281
Slackia	0 ± 0	0 ± 0	0.494
Staphylococcus	0.1 ± 0.1	0.1 ± 0	0.235
Streptococcus	1.1 ± 0.6	5 ± 3	0.152
Succinivibrio	0 ± 0	0 ± 0	0.494
Sutterella	0.2 ± 0.2	0.6 ± 0.4	0.819
Tannerella	0 ± 0	0 ± 0	0.254
Terrisporobacter	1.2 ± 0.7	1.8 ± 1.1	0.406
Trueperella	0 ± 0	0.6 ± 0.6	0.254
Turicibacter	0 ± 0	3.4 ± 3.3	0.287
Tyzzerella	1.5 ± 0.8	1 ± 0.6	0.75
Ureaplasma	0 ± 0	0 ± 0	0.254
Ursidibacter	0 ± 0	0.1 ± 0.1	0.254
Veillonella	1.2 ± 1.2	0 ± 0	0.281
Weissella	0 ± 0	0 ± 0	0.494
Others	0.2 ± 0.1	0.3 ± 0.1	0.085

Table S2. Relative abundance of fecal microbiome species present in healthy and sick dogs

Species	Healthy group	LM group	*p* Value
[Clostridium] colinum	0.4 ± 0.2	0.2 ± 0.2	0.706
[Clostridium] innocuum	0.6 ± 0.3	0.3 ± 0.2	0.522
[Clostridium] leptum	0 ± 0	0.1 ± 0	0.145
[Clostridium] methylpentosum	0 ± 0	0 ± 0	0.093
[Clostridium] spiroforme	1.5 ± 0.9	0.1 ± 0.1	0.042
[Eubacterium] tenue	0.2 ± 0.1	0.2 ± 0.1	0.426
[Ruminococcus] gnavus	4.6 ± 1.3	8.1 ± 4.3	0.928
[Ruminococcus] lactaris	0.2 ± 0.2	0 ± 0	0.494
[Ruminococcus] torques	0.1 ± 0	0.5 ± 0.4	0.731
Abyssivirga alkaniphila	0.2 ± 0.1	0.6 ± 0.4	0.216
Actinomyces weissii	0 ± 0	0 ± 0	0.74
Allisonella histaminiformans	0 ± 0	0 ± 0	0.254
Amedibacillus dolichus	0.5 ± 0.2	0.9 ± 0.4	0.272
Anaerobacterium chartisolvens	0 ± 0	0 ± 0	0.78
Anaerostipes caccae	0.1 ± 0.1	0 ± 0	0.119
Anaerotignum lactatifermentans	0.1 ± 0.1	0 ± 0	0.47
Arcanobacterium pinnipediorum	0 ± 0	0.1 ± 0.1	0.254
Bacteroides faecichinchillae	0.2 ± 0.2	0 ± 0	0.494
Bacteroides faecis	0.3 ± 0.2	0 ± 0	0.281
Bacteroides fragilis	5.2 ± 3.4	0.6 ± 0.3	0.964
Bacteroides pyogenes	0 ± 0	0 ± 0	0.254
Bacteroides stercorirosoris	0.1 ± 0.1	0 ± 0	0.494
Bacteroides stercoris	0.3 ± 0.3	0.5 ± 0.5	0.576
Bacteroides thetaiotaomicron	0.1 ± 0.1	0.4 ± 0.3	0.329
Bacteroides uniformis	0.5 ± 0.4	0.2 ± 0.2	0.889
Bacteroides xylanisolvens	0 ± 0	0.3 ± 0.2	0.265
Beduini massiliensis	0.1 ± 0.1	0 ± 0	0.78
Bifidobacterium catenulatum	0 ± 0	0 ± 0	0.494
Bifidobacterium longum	0 ± 0	0 ± 0	0.281
Bifidobacterium pullorum	0.2 ± 0.2	0 ± 0	0.281
Blautia argi	3.7 ± 1.8	2.9 ± 2.2	0.468
Blautia caecimuris	0.2 ± 0.1	0.5 ± 0.5	0.345
Blautia faecicola	0.1 ± 0	0 ± 0	0.619
Blautia glucerasea	0.1 ± 0.1	0.4 ± 0.4	0.957
Blautia hydrogenotrophica	0 ± 0	0.3 ± 0.3	0.254
Blautia marasmi	3 ± 1.3	6.7 ± 3.6	0.339
Blautia schinkii	3.5 ± 1.4	0 ± 0	0.016
Buchananella hordeovulneris	0 ± 0	0 ± 0	0.007

Bulleidia extructa	0 ± 0	0 ± 0	0.494
Butyribacter intestini	0 ± 0	0 ± 0	0.494
Butyricicoccus pullicaecorum	0.5 ± 0.3	1 ± 0.9	0.927
Campylobacter showae	0 ± 0	0.1 ± 0.1	0.254
Campylobacter upsaliensis	0.1 ± 0.1	0.1 ± 0.1	1
Canicola haemoglobinophilus	0 ± 0	0.2 ± 0.2	0.295
Caproiciproducens galactitolivorans	0.3 ± 0.3	0 ± 0	0.023
Catenibacterium mitsuokai	0 ± 0	0 ± 0	0.494
Cellulosilyticum lentocellum	0 ± 0	0 ± 0	0.422
Citrobacter braakii	0 ± 0	0.6 ± 0.6	0.74
Citrobacter europaeus	0 ± 0	0 ± 0	0.254
Citrobacter freundii	0 ± 0	0 ± 0	0.254
Citrobacter murliniae	0 ± 0	0.1 ± 0.1	0.082
Clostridioides difficile	1.2 ± 0.5	2.2 ± 1.3	0.891
Clostridium baratii	0 ± 0	0 ± 0	0.254
Clostridium paraputrificum	0.4 ± 0.2	0.9 ± 0.4	0.363
Clostridium perfringens	1.5 ± 1.2	2.7 ± 1.4	0.82
Clostridium saccharobutylicum	0.9 ± 0.7	0.2 ± 0.2	0.537
Clostridium saudiense	0.4 ± 0.2	0.9 ± 0.4	0.356
Clostridium septicum	0.1 ± 0	0.4 ± 0.3	0.476
Clostridium tertium	0 ± 0	0 ± 0	0.619
Clostridium thiosulfatireducens	0 ± 0	0 ± 0	0.254
Collinsella intestinalis	0.4 ± 0.2	0 ± 0	0.456
Coprobacter fastidiosus	0 ± 0	0.1 ± 0.1	0.254
Corynebacterium amycolatum	0.5 ± 0.4	4.3 ± 1.5	0.016
Corynebacterium canis	0 ± 0	0 ± 0	0.74
Corynebacterium freiburgense	0 ± 0	0.2 ± 0.2	0.534
Corynebacterium lowii	0 ± 0	0.9 ± 0.9	0.136
Corynebacterium mastitidis	0.6 ± 0.4	2.7 ± 2.2	0.356
Corynebacterium matruchotii	0 ± 0	0.1 ± 0.1	0.169
Corynebacterium mustelae	0 ± 0	0 ± 0	0.329
Corynebacterium tuberculostearicum	0 ± 0	0 ± 0	0.494
Desulfovibrio intestinalis	0 ± 0	0 ± 0	0.74
Dialister succinatiphilus	0 ± 0	0 ± 0	0.494
Eggerthella lenta	0 ± 0	0.2 ± 0.2	0.842
Enterobacter cloacae	0 ± 0	0 ± 0	0.494
Enterobacter mori	0 ± 0	0 ± 0	0.254
Enterocloster aldenensis	0 ± 0	0 ± 0	0.281
Enterocloster clostridioformis	0 ± 0	0.1 ± 0	0.256
Enterococcus avium	0 ± 0	0.1 ± 0.1	0.494
Enterococcus faecalis	0.1 ± 0	0.3 ± 0.1	0.059

Enterococcus gallinarum	0 ± 0	0.1 ± 0.1	0.082
Enterococcus hirae	0.1 ± 0.1	2.3 ± 1.5	0.252
Enterococcus raffinosus	0 ± 0	0 ± 0	0.494
Enterococcus saigonensis	0 ± 0	0 ± 0	0.483
Erysipelatoclostridium ramosum	0.7 ± 0.2	0.5 ± 0.3	0.255
Escherichia fergusonii	15.2 ± 4.7	19.3 ± 6	0.365
Faecalibacterium prausnitzii	0.1 ± 0.1	0 ± 0	0.78
Faecalimonas umbilicata	4.3 ± 1.9	2.9 ± 1	1
Finegoldia magna	0 ± 0	0.5 ± 0.4	0.265
Flavonifractor plautii	0.2 ± 0.1	0.2 ± 0.1	0.236
Flintibacter butyricus	0 ± 0	0 ± 0	0.517
Fournierella massiliensis	0 ± 0	0 ± 0	0.28
Frederiksenia canicola	0 ± 0	0 ± 0	0.494
Fusobacterium gastrosuis	0 ± 0	0.4 ± 0.4	0.254
Fusobacterium mortiferum	7.9 ± 4.5	6.3 ± 4.1	1
Fusobacterium nucleatum	0 ± 0	0 ± 0	0.082
Fusobacterium perfoetens	1.2 ± 0.7	1.6 ± 1.6	1
Fusobacterium russii	0 ± 0	0 ± 0	0.254
Fusobacterium simiae	0 ± 0	0 ± 0	0.254
Fusobacterium varium	0 ± 0	0 ± 0	0.494
Gemella palaticanis	0 ± 0	0 ± 0	0.254
Gleimia coleocanis	0 ± 0	0 ± 0	0.082
Gluceribacter canis	2.3 ± 0.6	0.9 ± 0.8	0.118
Haemophilus pittmaniae	0 ± 0	0 ± 0	0.254
Helicobacter bilis	0 ± 0	0.5 ± 0.5	0.254
Helicobacter canicola	0 ± 0	0.1 ± 0.1	0.74
Helicobacter canis	3.7 ± 3.7	0 ± 0	0.281
Kineothrix alysoides	1.4 ± 0.3	0.7 ± 0.6	0.036
Klebsiella oxytoca	0 ± 0	0 ± 0	0.74
Klebsiella pneumoniae	0.1 ± 0.1	0.3 ± 0.3	0.302
Klebsiella variicola	0 ± 0	0.1 ± 0.1	0.051
Lacrimispora amygdalina	0 ± 0	0 ± 0	0.494
Lactobacillus rogosae	0 ± 0	0 ± 0	0.494
Lactonifactor longoviformis	0 ± 0	0 ± 0	0.254
Lawsonella clevelandensis	0 ± 0	0.2 ± 0.2	0.254
Ligilactobacillus salivarius	0 ± 0	0.3 ± 0.3	0.254
Massilimicrobiota timonensis	0 ± 0	0.1 ± 0.1	0.74
Mediterranea massiliensis	0.9 ± 0.9	0 ± 0	0.494
Mediterraneibacter glycyrrhizinilyticus	0.2 ± 0.1	0.4 ± 0.4	0.216
Megamonas funiformis	1.8 ± 1.6	0 ± 0	0.534
Morganella morganii	0 ± 0	0.2 ± 0.2	0.265

Natranaerovirga pectinivora	0 ± 0	0 ± 0	0.254
Novisyntrophococcus fermenticellae	0 ± 0	0 ± 0	0.889
Odoribacter splanchnicus	0 ± 0	0 ± 0	0.494
Paeniclostridium sordellii	0 ± 0	0.2 ± 0.2	0.254
Parabacteroides distasonis	0 ± 0	0 ± 0	0.494
Parabacteroides merdae	5.4 ± 4.3	0.2 ± 0.2	0.534
Paraprevotella xylaniphila	0 ± 0	0 ± 0	0.494
Parasutterella secunda	0.1 ± 0.1	0 ± 0	0.494
Pasteurella canis	0 ± 0	0.2 ± 0.2	0.254
Peptacetobacter hiranonis	2.7 ± 2.6	0 ± 0	0.78
Peptoniphilus phoceensis	0 ± 0	0.2 ± 0.2	0.254
Peptostreptococcus canis	0 ± 0	0.3 ± 0.2	0.046
Phascolarctobacterium faecium	0 ± 0	0 ± 0	0.868
Phascolarctobacterium succinatutens	0.1 ± 0.1	0 ± 0	0.494
Phocaeicola coprocola	1 ± 1	0.3 ± 0.3	0.756
Phocaeicola coprophilus	0.2 ± 0.2	0 ± 0	0.494
Phocaeicola plebeius	0.1 ± 0.1	0.1 ± 0.1	0.619
Phocaeicola vulgatus	9.9 ± 5.9	0.7 ± 0.6	0.551
Phocea massiliensis	0 ± 0	0.1 ± 0.1	0.295
Porphyromonas cangingivalis	0 ± 0	1.7 ± 1.7	0.265
Porphyromonas gingivicanis	0 ± 0	0.9 ± 0.9	0.254
Prevotella copri	0 ± 0	0 ± 0	0.254
Prevotellamassilia timonensis	0.4 ± 0.4	0 ± 0	0.494
Proteus mirabilis	0.2 ± 0.1	0.6 ± 0.2	0.042
Pseudoflavonifractor capillosus	0 ± 0	0 ± 0	0.281
Pseudomonas deceptionensis	0 ± 0	0 ± 0	0.254
Romboutsia sedimentorum	0.1 ± 0	0.9 ± 0.4	0.236
Romboutsia timonensis	0 ± 0	0 ± 0	0.329
Roseburia intestinalis	0.4 ± 0.3	0 ± 0	0.033
Ruthenibacterium lactatiformans	0 ± 0	0 ± 0	0.254
Schaalia canis	0 ± 0	0.1 ± 0.1	0.059
Sellimonas intestinalis	0 ± 0	0 ± 0	0.281
Shigella boydii	0.4 ± 0.2	0 ± 0	0.281
Slackia faecicanis	0 ± 0	0 ± 0	0.494
Staphylococcus aureus	0 ± 0	0 ± 0	0.494
Staphylococcus intermedius	0.1 ± 0.1	0.1 ± 0	0.776
Streptococcus alactolyticus	0 ± 0	0.6 ± 0.6	0.254
Streptococcus canis	0 ± 0	0.1 ± 0	0.494
Streptococcus fryi	0 ± 0	0 ± 0	0.026
Streptococcus lutetiensis	1.1 ± 0.6	4.2 ± 2.5	0.537
Streptococcus minor	0 ± 0	0 ± 0	0.082

Streptococcus salivarius	0 ± 0	0 ± 0	0.494
Succinivibrio dextrinosolvens	0 ± 0	0 ± 0	0.494
Sutterella massiliensis	0.1 ± 0.1	0.4 ± 0.4	1
Sutterella stercoricanis	0.2 ± 0.2	0.2 ± 0.2	0.868
Sutterella wadsworthensis	0 ± 0	0 ± 0	0.494
Tannerella forsythia	0 ± 0	0 ± 0	0.254
Terrisporobacter petrolearius	1.2 ± 0.7	1.8 ± 1.1	0.406
Trueperella bernardiae	0 ± 0	0.6 ± 0.6	0.254
Turicibacter sanguinis	0 ± 0	3.4 ± 3.3	0.287
Tyzzerella nexilis	1.2 ± 0.6	0.8 ± 0.6	0.927
Ureaplasma parvum	0 ± 0	0 ± 0	0.254
Ursidibacter maritimus	0 ± 0	0.1 ± 0.1	0.254
Veillonella atypica	1.2 ± 1.2	0 ± 0	0.494
Veillonella dispar	0 ± 0	0 ± 0	0.494
Others	0.2 ± 0.1	0.3 ± 0.1	0.014
